# Psychological Impact of Infection Fear on Healthcare Provider during the COVID-19 Pandemic: Considerations for Health Promotion

**DOI:** 10.34172/hpp.44248

**Published:** 2026-06-06

**Authors:** Zohre Ahmadi, Farkhondeh Amin Shokravi, Hormoz Sanaeinasab, Aliakbar Rasekhi

**Affiliations:** ^1^Department of Health Education and Health Promotion, Faculty of Medical Sciences, Tarbiat Modares University, Tehran, Iran; ^2^Department of Public Health, Faculty of Medical Sciences, Islamic Azad University, Tehran, Iran; ^3^Department of Health Promotion, Iranian Academy of Medical Sciences, Tehran, Iran; ^4^Department of Biostatistics, Faculty of Medical Sciences, Tarbiat Modares University, Tehran, Iran

**Keywords:** COVID-19, Fear, Healthcare workers, Infection, Psychological impact, Health promotion, Pandemic, Well-being

## Abstract

**Background:**

The COVID-19 pandemic has imposed substantial psychological pressure on healthcare workers (HCWs), largely due to fear of infection. Understanding the factors associated with this fear is essential for designing effective health promotion interventions. This study aimed to identify determinants of fear of COVID-19 infection among HCWs in Qom Province, Iran.

**Methods:**

This cross-sectional study was conducted in 2022 among 385 healthcare workers, including physicians, dentists, nurses, and health specialists. Data were collected using the standard S19-HCPS questionnaire. Fear of COVID-19 infection was considered the primary outcome measure. Data were analyzed using SPSS software, and multiple linear regression analysis was applied to identify significant predictors.

**Results:**

Approximately 50% of participants were male. Multiple linear regression analysis indicated that protective behaviors (β=−0.087, *P*=0.015), attitude toward COVID-19 (β=−0.118, *P*<0.001), perceived health policies (β=−0.773, *P*<0.001), and desire to provide patient care (β=−0.114, *P*<0.001) were significant predictors of fear of COVID-19 infection. These variables collectively explained 66.1% of the variance in fear scores.

**Conclusion:**

Fear of COVID-19 infection remains a significant concern among healthcare workers and may adversely affect their well-being and professional performance. Strengthening supportive health policies, promoting protective behaviors, and addressing attitudinal factors should be prioritized to reduce infection-related fear and enhance mental well-being among HCWs during ongoing and future public health emergencies.

## Introduction

 The novel coronavirus first emerged in the city of Wuhan, China, and due to its rapid and widespread global transmission, the World Health Organization (WHO) declared it a pandemic and a public health emergency of international concern.^1^ Based on the latest WHO report, until 16 November 2023, globally 773 million confirmed cases and 6,980,000 deaths were occurred. In Iran, the cumulative number of confirmed cases and deaths reported were equal to 7,623,000 and 146,000, respectively.^2^ Given the number of deaths caused by the coronavirus COVID-19, anxiety, worry, and general fear have increased among people in many regions. Fear and anxiety (as the consequences of epidemics) are understandable, but may lead to psychosocial challenges such as stigma and social discrimination against the infected individuals.^3,4^

 The COVID-19 pandemic has profoundly affected public mental health, extending beyond physical health concerns to contribute to a wide range of psychological disorders. So, it is critical to implement targeted psychological interventions to support the well-being of vulnerable populations during the COVID-19 crisis.^5^ The COVID-19 pandemic has had a profound psychological impact, prompting extensive research. A comprehensive study involving 68 research projects from 19 countries investigated the psychological effects of COVID-19.^6^ The results revealed a high prevalence of anxiety and depression within the general population. Women, younger individuals, rural residents, and those with lower socioeconomic status were more prone to anxiety compared to men, older individuals, urban residents, and those with higher socioeconomic status, respectively. Furthermore, the higher risk of COVID-19 infection—such as suspected or confirmed cases, residing in heavily affected areas, or having pre-existing physical or mental health conditions—along with prolonged media exposure, was found to be associated to increased rates of anxiety and depression.^7^ Approximately one in three adults experienced psychological distress related to COVID-19.^8^ According to the WHO, some HCPs are rejected and avoided by their families or friends due to fear or stigma associated with the coronavirus.^9^

 The COVID-19 pandemic has negatively affected critical care healthcare professionals (HCPs) in terms of their work experience and well-being. Fear of contracting COVID-19 also exists among HCPs, but their understanding and knowledge of disease leads to a willingness to care for COVID-19 patients.^10^ Frontline HCPs face significant psychological challenges during disease outbreaks. These may include fear of infection, potential quarantine, worry about infecting loved ones, caring for sick colleagues, and social stigma.^11^ predominantly, the psychological issues confronting the HCPs may include high levels of depression, fear, anxiety, insomnia, and distress.^12^ Two-thirds of HCPs experienced moderate levels of fear and anxiety, with female participants reporting higher levels. Social distancing is significantly associated to social well-being, highlighting the need for policies that enhance social support.^13^ Health care workers and public service providers have exhibited elevated levels of post-traumatic stress disorder (PTSD) symptoms, anxiety, and depression during the COVID-19 pandemic. Among health care workers, those in direct contact with COVID-19 patients have demonstrated significantly higher rates of PTSD symptoms and depression compared to those with indirect patient care responsibilities.^14^ A meta-analysis reviewed 65 studies that assessed the prevalence of psychological issues such as anxiety, depression, PTSD, and stress among HCPs during the COVID-19 pandemic.^15^ In this review the higher psychological consequences were reported among healthcare personnel, particularly women, nurses, and frontline responders. Another notable finding was the pandemic’s pervasive impact on all aspects of their life.^16^ Another meta-analysis study have identified a high prevalence of moderate depression, anxiety and PTSD among health care workers during the COVID-19 pandemic.^17^

 The outbreak of COVID-19 can be considered an important experience of a bio-disaster resulting in a significant rate of psychiatric problems in HCPs. Detecting and managing concerns and reducing infection-related embarrassment/stigma are essential for improving HCPs’ mental health.^18^ To do so, organizing mental health workshops and programs to address fear and anxiety may enable HCPs to cope with the challenges of pandemics, particularly its associated anxiety and fear.^19^ Mental health promotion strategies, such as physical activity, relaxation activities, recreational activities, healthy diet, adequate water intake, breaks between work shifts, maintenance of remote social contacts, and verbalization of feelings/emotions, are crucial to reduce HCPs’ stress, anxiety and depression symptoms during the COVID-19.^20^

 During the pandemic, fear significantly impacted nurses’ health-promoting and protective behaviors. A study of 301 nurses at a university hospital indicated that higher fear levels were associated with reduced effectiveness in health promotion activities, underscoring the need for interventions to mitigate fear and support nurses in maintaining effective health promotion efforts.^21^ Based on the findings of a systematic review on the fear of infection and transmission among healthcare workers during the quarantine for COVID-19, the quarantine, due to the risk of infection, generated significant psychological stress among healthcare workers. The results indicated that the healthcare workers, in the long term, exhibited symptoms of depression, post-traumatic stress, and alcohol misuse.^22^ National and local health systems should consider the needs of healthcare providers when designing support initiatives.^23,24^ Another systematic review reported that the prevalence of anxiety among HCPs during the pandemic ranged from 14.5% to 44.6%, while depression rates varied between 8.9% and 50.4%.^25^

 Understanding and addressing the psychosocial factors associated to the pandemic among HCPs are crucial for developing strategies to enhance health promotion efforts during ongoing and future public health crises. Fear, as a powerful negative emotion, significantly impacted HCPs during the COVID-19 pandemic, often led to avoidance behaviors due to perceived risks. HCPs experience higher rates of anxiety and fear compared to others. While fear is a natural reaction, courage involves trusting proven infection prevention practices to deliver high-quality care in the safest possible environment. Choosing courage is essential for HCPs to overcome fear and maintain effective healthcare delivery.^26,27^

 In this study, we aim to investigate the psychological impacts of fear of COVID-19 infection among HCPs during the pandemic in Qom County, Iran.

## Material and Methods

###  Study design and setting

 This cross-sectional study was conducted to investigate stigma and factors associated with fear of COVID-19 infection among healthcare providers (HCPs) in Qom County, Iran. Data were collected from HCPs working in Qom health centers who were providing services to patients with COVID-19 during the period from 2022 to 2023.

###  Participants and sampling

 The study population consisted of healthcare providers who had direct contact with patients with COVID-19 in Qom health centers. A list of all eligible HCPs was obtained from the administrative records of the health centers. Each eligible participant was assigned a unique identification number. Simple random sampling was then performed using a computer-generated random number table to select participants from the sampling frame. This randomization process ensured an unbiased and representative sample of the target population.

###  Sample size calculation

 The sample size was calculated using Cochran’s formula, assuming a prevalence of 50% for fear-related outcomes due to the lack of prior evidence, with a 95% confidence level and a margin of error of 5%. This conservative estimate was used to ensure sufficient statistical power for assessing associations and correlations among study variables. Given that simple random sampling was applied, the design effect was assumed to be 1. Based on these assumptions, the minimum required sample size was calculated as 385 participants.

###  Inclusion and exclusion criteria

 The inclusion criteria were: (1) being a healthcare provider working in Qom health centers and (2) having direct contact with patients with COVID-19. The exclusion criteria included: (1) having physical or mental health conditions that could interfere with participation in the study and (2) incomplete questionnaire responses.

###  Measures

 The Stigma of COVID-19 Healthcare Providers Scale (S19-HCPS) was used to assess stigma and related factors among healthcare providers. S19-HCPS stands for Stigma of COVID-19 Healthcare Providers Scale. The questionnaire was adapted and translated into Persian following a standard forward–backward translation procedure. The instrument consists of two sections. The first section includes demographic characteristics (items 1–6), covering gender, age, occupation, and years of work experience. The second section comprises 30 items organized into six subscales assessing different dimensions of stigma and related constructs: (1) Fear of Infection (7 items; e.g., “I am afraid of contracting COVID-19 while working with patients”), (2) Protective Behaviors (5 items; e.g., “I frequently use personal protective equipment to avoid infection”), (3) Attitude (6 items; e.g., “I believe healthcare providers should avoid treating COVID-19 patients to stay safe”), (4) Being Judged by Others (5 items; e.g., “I feel that others judge me negatively for working with COVID-19 patients”), (5) Health Policies (4 items; e.g., “I believe workplace policies adequately protect healthcare providers from infection”), and (6) Willingness to Care for Patients (3 items; e.g., “I am willing to continue providing care to COVID-19 patients”). All items were scored on a five-point Likert scale ranging from 0 (Strongly disagree) to 4 (Strongly agree). Subscale scores were calculated by summing item scores within each domain, with higher scores indicating greater intensity of the measured construct. The internal consistency of the Persian version of the questionnaire was assessed using Cronbach’s alpha coefficient, which was 0.78, indicating acceptable reliability. In the original validation study, the Cronbach’s alpha coefficient for the total scale was reported to be greater than 0.70.^28^

###  Data collection

 Before data collection, participants were informed about the study objectives and procedures. Data were collected using a self-administered questionnaire under the supervision of a trained interviewer, who provided clarification when necessary. Participation was voluntary, and no personally identifiable information was collected. Participants were assured that all data would be kept confidential and used solely for research purposes.

###  Statistical analysis

 Data analysis was performed using IBM SPSS Statistics for Windows, version 26.0 (IBM Corp., Armonk, NY, USA). Descriptive statistics, including mean, standard deviation, frequency, and percentage, were used to summarize the data. Inferential analyses included independent t-tests, one-way analysis of variance (ANOVA), Pearson correlation coefficients, and multiple linear regression analysis to examine associations between stigma-related outcomes and explanatory variables. All statistical tests were two-tailed, and a p-value of less than 0.05 was considered statistically significant. Where appropriate, 95% confidence intervals were reported. For the multiple linear regression analyses, variables that showed statistically significant associations in the bivariate analyses were entered into the regression models. Prior to model fitting, the assumptions of linear regression were assessed. The normality of residuals was evaluated using histograms and normal probability plots, and homoscedasticity was assessed through visual inspection of residual scatterplots. Multicollinearity was examined using variance inflation factor (VIF) and tolerance values, with VIF values less than 5 indicating the absence of multicollinearity. The independence of errors was assessed using the Durbin–Watson statistic. No significant violations of regression assumptions were observed; therefore, no remedial measures were required.

## Results

 A total of 385 healthcare providers (HCPs) from healthcare centers in Qom participated in the study. Approximately half of the participants were men (50.1%, 95% CI: 45.1–55.1). The majority of participants were aged 31–40 years (48.8%, 95% CI: 43.8–53.8), and the largest occupational group was health workers (38.8%, 95% CI: 33.9–43.7). Nearly half of the participants had 6–10 years of job experience (47.3%, 95% CI: 42.3–52.3) (Table 1).

**Table 1:** Demographic characteristics of the study participants (n = 385)

**Variable**	**Category**	**Frequency**	**Percentage **
Gender	Man	193	50.1
	Female	192	49.9
Age	20-30	128	33.2
	31 – 40	188	48.8
	41-50	65	16.9
	+ 51	4	1
Occupation	Physician	92	23.9
	Nurse	99	25.7
	Dentist	37	9.6
	Health worker (e.g., public health staff)	149	38.8
	Other health professionals	8	2.1
Job experience (years)	≤ 5	129	33.5
	6–10	182	47.3
	11–20	67	17.4
	≥ 21	7	1.8
		383	100

 Descriptive statistics for the study variables are presented in Table 2. Based on the study findings, the mean score for the “Being judged by others” dimension was the highest (3.88 ± 1.18), while the mean score for the “Health policies” dimension was the lowest (2.86 ± 2.18) among the study population.

**Table 2:** Mean and standard deviation of the S19-HCPS domains

**Variables**	**Mean±standard deviation**	**The highest score **	**The lowest score **	**Percentage of score obtained**
Fear of infection	6.77 ± 3. 40	19	0	35.6
Protective behaviors	3.52 ± 1.97	8	0	44
Attitude	7.01 ± 2.14	13	0	53.9
Being judged by others	3.88 ± 1.18	8	0	48.5
Health Policies	2.86 ± 2.18	9	0	31.7
Willingness to care for the patient	5.1 ± 1.50	9	1	56.7

 Pearson correlation analysis showed statistically significant correlations between most variables (*P* < 0.05). However, the associations between attitude and protective behaviors, as well as between attitude and being judged by others, were not statistically significant (*P* > 0.05) (Table 3).

**Table 3:** Pearson Correlation Coefficients of the studied domains with Protective behaviors

**Variables**	**Protective Behaviors**	**Attitude**	**Being Judged by Others**	**Health Policies**	**Willingness to Care for Patients**	**Fear**
Protective Behaviors	1					
Attitude	r = -0.280, *P* < 0.280	1				
Being Judged by Others	r = 0.554, *P* < 0.001	r = 0.305, *P* < 0.001	1			
Health Policies	r = 0.277, *P* < 0.001	r = 0.221, *P* < 0.001	r = 0.305, *P* < 0.001	1		
Willingness to Care for Patients	r = 0.465, *P* < 0.000	r = 0.196, *P* < 0.000	r = 0.350, *P* < 0.000	r = 0.159, *P* < 0.002	1	
Fear	r = 0.176, *P* < 0.001	r = 0.332, *P* < 0.000	r = 0.320, *P* < 0.000	r = 0.794, *P* < 0.001	r = 0.238, *P* < 0.001	1

 Regression analysis revealed that the variables of protective behaviors, attitude, policy, and desire for patient care were the most significant predictors of anxiety or fear, accounting for 66.1% of the variance (Table 4). One-way ANOVA revealed significant differences in fear (*P* < 0.001), protective behaviors (*P* < 0.001), and health policies (*P* < 0.001) by occupation. Additionally, independent samples t-test results indicated significant differences in protective behaviors (*P* < 0.001) and being judged by others (*P* = 0.045) by level of education.

**Table 4:** Multiple linear regression analysis of predictors of anxiety and fear related to stigma

**Predictor**	**B**	**S. E**	**β**	**T**	***P*** **-value**
Protective behaviors	-0.15	0.062	-0.087	-2.43	0.015
Attitude	0.188	0.053	0.118	3.52	< 0.0001
Health policies	1.20	0.050	0.773	24.24	< 0.0001
Willingness to care for the patient	0.259	0.083	0.114	3.10	0.002
R = 0.81 R^2^= 0.665 ADJ.R^2^ = 0.661					

## Discussion

 The present study aimed to investigate the factors associated with fear of infection among HCPs in Qom County, Iran during the COVID-19 pandemic. The findings indicated that fear of infection, protective behaviors, attitudes toward the disease, being judged by others, health policies, and willingness to care for patients were significant predictors of stigma. Similar with our findings, a previous study reported that the fear of contracting illness and the fear of transmitting infections and diseases, including COVID-19, have sometimes led to nurses and HCPs leaving their jobs during the pandemic.^29^ Our study also found that 35.6% of healthcare personnel fear contracting COVID-19. Consistent with our results, several studies have highlighted the fear of infection as a major contributing factor to stigma among HCPs.^30^ Cassiani-Miranda et al. reported a significant link between fear of COVID-19 and stigma, underscoring the public health implications of fear-driven social attitudes.^31^ Another study indicated that hospital work, particularly caring for COVID-19 patients, was a significant risk factor for heightened anxiety levels among medical staff.^32^ Based on the above results, it appears that issues related to stigma, fear, anxiety, and other psychological matters among HCPs during the pandemic were closely intertwined. An Ethiopian study found associations between the level of perceived vulnerability to COVID-19 and inconsistent adherence to wearing gloves and masks among health professionals, suggesting a need for better support to enhance compliance.^33^ In contrast, our findings focused on the psychological impact of fear and stigma, identifying their effects on healthcare providers’ mental health. While both studies address psychological challenges during COVID-19, the Ethiopian study prioritizes risk perception interventions, whereas our findings emphasizes mental health promotion interventions aiming at controlling fear and stigma among the population. In the face of the critical situation caused by the COVID-19 pandemic, HCPs and medical staff in hospitals were on the frontline of the fight against the disease.^34,35,36^ For most health service providers during crises, many issues are stressful, including: the risk of contracting the disease, high workload, witnessing the suffering and pain of the patients and their families, separation from family, difficult decisions in life-and-death situations, and providing support in medical care.^37^

 Studies on the fear of COVID-19 among HCPs have also been conducted in various other countries around the world. In a study by Khanal et al^38^ the nursing home professionals showed high levels of fear of contagion and secondary traumatic stress when faced with the COVID-19 crisis.^39^ Almarghlani et al. (2022) also found that their participants were fearful of the COVID-19 virus and the possibility of being transmitters, but a majority were confident in their ability to treat COVID-19-positive patients,^40^ which were similar to those found in the present study. Fear and avoidance of HCPs is a widespread, under-recognized problem during the COVID-19 pandemic.^39,41^ It seems that the societal phobia towards them is one of the reasons for the fear experienced by healthcare providers. Fear can arise from various factors such as the unknown nature of the coronavirus during the pandemic, its contagiousness, morbidity and mortality rates, and even fear of transmitting the disease to friends and acquaintances. The findings of our study indicated a significant correlation between work experience and both the fear of COVID-19 infection and the adoption of protective behaviors among HCPs. Similarly, Alnazly et al. reported that work experience was significantly associated with psychological distress, including fear of COVID-19 infection, among nurses.^42^ The dissimilarity between our findings and those of Alnazly et al. may be attributed to variations in the average age of study participants, cultural differences, or the time of conducting the studies. The COVID-19 pandemic has placed immense stress on healthcare systems globally, revealing significant weaknesses in supporting HCPs. A major concern is the intense fear and anxiety faced by HCPs due to resource shortages, high exposure risks, and overwhelming workloads. This fear has often resulted in lapses in standard preventative measures, raising the risk of infection for both staff and patients.^26,43^ A qualitative study from Sweden highlighted that the fear of HCPs was intensified by confusion over personal protective equipment, infection prevention and control (PPE/IPC) guidelines, and inconsistent leadership during the early stages of the pandemic.^44^ The authors suggested that prior experience with infectious diseases and clear and consistent communication from leadership can bolster confidence and reduce fear among HCPs. In the study conducted by Luceno-Moreno et al. long working hours in contact with COVID-19 patients among nurses led to a greater fear of infection and transmission.^45^ The findings of another study revealed that the existence or non-existence of protective rules and health authority support had played a role in HCPs’ fear.^46^ Our results also indicated a significant association between protective behaviors and the experience of being judged by others. This finding is consistent with those of previous studies that showed higher knowledge and risk perception may often lead to both more cautious behavior and, paradoxically, social stigma from others who misunderstood such caution.^47^ This paradox may further intensify the psychological burden on HCPs.

 Based on the results of the present study, age and gender had no statistically significant relationship with any of the studied variables, which was similar to those reported by Abid et al,^48^ suggesting that such factors may not play a substantial role in influencing stigma or fear in this context. One possible explanation is that during a global health crisis, like the COVID-19 pandemic, the perceived threat and associated psychological responses—such as fear of infection or stigma may transcend basic demographic boundaries, affecting HCPs more uniformly regardless of their age or gender. It is also possible that occupational roles, organizational policies, and personal exposure to COVID-19 patients had a strong and immediate influence on HCPs’ psychological outcomes, overshadowing the impact of static demographic characteristics. This lack of association could also be associated to cultural and contextual factors specific to the region studied, such as similar workplace expectations or access to information and support across different age groups and genders. It might also reflect limitations in sample size or variability within subgroups, reducing the statistical power to detect subtle differences. Future studies with larger and more diverse samples, or those focusing specifically on age- and gender-based experiences, may provide greater insight into these dynamics

 Considering that there was a moderate correlation between fear of COVID-19 and health protective behaviors, it is recommended to have a focus on the interventions aimed at reducing fear of COVID-19.^21,49,50^ In Qom Province, various cultural factors may shape HCPs fear of infection during the COVID-19 pandemic. The city’s religious significance fosters a strong sense of duty toward collective well-being, which may intensify anxiety due to close patient contact. While community support can boost morale, it may also create pressure to fulfill heroic expectations, heightening stress. Cultural stigma surrounding illness may further contribute to HCPs’ fear of both infection and social judgment. Additionally, traditional beliefs and inconsistent communication may cause confusion about preventive measures, undermining confidence in safety protocols. Limited trust in healthcare systems and access to resources can also influence HCPs’ sense of preparedness and mental well-being. Understanding these cultural dynamics is essential for designing effective, context-sensitive health interventions.

## Limitations

 This study has several limitations that should be considered when interpreting the results. First, the cross-sectional design precludes establishing causality among the studied variables, such as the relationships between stigma-related factors and fear of infection. Second, the use of self-reported data via the S19-HCPS questionnaire may introduce response bias, as participants’ answers could be influenced by social desirability or recall inaccuracies. Third, the study was conducted in Qom health centers, which may limit the generalizability of the findings to other regions or healthcare settings with different cultural or operational contexts. Finally, while the sample size was sufficient for the study’s objectives, the inclusion of only HCPs with direct contact with COVID-19 patients may not capture the experiences of other healthcare professionals indirectly involved in COVID-19 care. Future studies could employ longitudinal designs or include diverse populations to further explore these associations.

## Conclusion

 COVID-19 poses significant challenges to the mental health of HCPs, as their fear of infection impacts both their performance and quality of life. These findings underscore the need to address social stigma, which arises from fear of infection. With the ongoing emergence of new COVID-19 variants, prioritizing the mental well-being of HCPs seems to be crucial for an effective healthcare response during epidemics.

## Competing Interests

 The authors declare that they have no competing interests.

## Ethical Approval

 This study was approved by the Research Ethics Committee of Tarbiat Modares University (IR.MODARES.REC.1401.193; approval date: 2022-12-24). All procedures were conducted in accordance with the ethical standards of the institutional research committee and the Declaration of Helsinki. Written informed consent was obtained from all participants prior to data collection. Participants were assured of the confidentiality of their data and their right to withdraw from the study at any stage without any consequences.
